# Studies on the Mechanism of Glutamate Metabolism in NTG-Induced Migraine Rats Treated with DCXF

**DOI:** 10.1155/2019/1324797

**Published:** 2019-12-31

**Authors:** Ni Ni, Qingqing Wang, Xiao Lin, Yanlong Hong, Yi Feng, Lan Shen

**Affiliations:** ^1^School of Pharmacy, Shanghai University of Traditional Chinese Medicine, Shanghai 201203, China; ^2^Engineering Research Center of Modern Preparation Technology of Traditional Chinese Medicine of Ministry of Education, Shanghai University of Traditional Chinese Medicine, Shanghai 201203, China; ^3^Shanghai University of Traditional Chinese Medicine, Shanghai Collaborative Innovation Center for Chinese Medicine Health Services, Shanghai 201203, China

## Abstract

**Objective:**

To explore the mechanism of the antimigraine effect by active components extracted from the Dachuanxiong prescription (DCXF), nitroglycerin- (NTG-) induced migraine rats were used to detect the change of glutamate metabolism and the overall metabolic profile at different time points in the serum and Trigeminocervical complex(TCC) samples.

**Method:**

The biological samples that were obtained at 30 minutes, 60 minutes, and 90 minutes after model establishment or drug administration were tested by GC-TOF-MS. Then, real-time PCR and western blot were applied to detect changes in the expression of some substances involved in glutamate metabolism.

**Result:**

DCXF could improve the metabolic profile of serum and TCC in migraine rats and showed the time trend of treatment, mainly involved by amino acid metabolism (glutamate, aspartic acid, and alanine metabolism). In addition, DCXF could increase the expressions of GS at 60 min and 90 min and EAAT1 at 90 min. The results of GS protein were similar to that of mRNA.

**Conclusion:**

The antimigraine effect of DCXF could be achieved by improving the metabolic profile and increasing the expressions of GS and EAAT1 to promote the glutamate cycle of TCC and serum samples in NTG-induced migraine rats to a certain extent.

## 1. Introduction

Migraine is a common, complex neurological disorder with high prevalence. National Health and Nutritional Research shows that migraine and severe headaches have affected nearly 72 million Americans (22.7% of the total population) [1]. A population-based door-to-door survey revealed that the 1-year prevalence of primary headache disorders in China was 23.8% with limited reach of headache services and high rates of underdiagnosis and misdiagnosis, causing enormous influence on the quality of life as well as a great social medical burden [2, 3]. Not only that, according to extensive research over the last decades, it was found that migraine may be associated with increased risk for other physical and psychiatric comorbidities, such as depression [4–7], epilepsy [8, 9], and ischemic stroke [10, 11], in the pathogenesis of a certain link. Considering the migraine as the most common disabling neurological disease, its treatment status is often unsatisfactory because of the lack of available acute and preventive therapies.

Dachuanxiong prescription is a classic and famous traditional Chinese medicine prescription with a long history to treat migraine, containing two herbal medicines: Gastrodia (*Gastrodia elata*Bl) and Chuanxiong (*Ligusticum chuanxiong Hort*). In the theory of the traditional Chinese medicine (TCM), Dachuanxiong prescription can activiate blood, dissolve stasis, and calm liver wind to have a good effect on migraine. The active components (DCXF) were extracted from the Dachuanxiong prescription by modern technology in the previous studies, retaining good efficacy of treatment of migraine [12]. Studies have reported that the DCXF showed significant antimigraine effect in decreasing the content of 5-hydroxytryptamine (5-HT) in the modelling rats induced by nitroglycerin (NTG) [13]. However, existing studies are mainly based on 5-HT and calcitonin gene-related peptide (CGRP). Little is known about the amino acid metabolism in the respect of drugs on migraine. Whether there are other ways to treat migraine by DCXF remains a question.

The emerging field of metabolomics provides a promising method to explore the therapeutic effect of drugs, especially the TCM, and to identify biomarkers that are closely related to the disease, establish new treatments, and toxicology [14, 15]. Tao et al. had investigated the metabolic profiles of serum in coronary heart disease patients by nontargeted metabolomics analysis to detect differential metabolites between the Xuefu Zhuyu decoction group and placebo group [16].

In the central nervous system (CNS), there are a large number of free amino acids. These amino acids, in addition to be the constituent units of proteins, also act as a neurotransmitter to play a role in signal transduction and participate in metabolic pathways [17]. Glutamate (Glu) has been demonstrated to be involved in migraine attacks. According to plenty of clinical studies and animal experiments, Glu works by the following ways [18–21]: (1) involving in the induction and maintanance of central sensitization in the pain state; (2) inducing Cortical Spreading Depression (CSD), which is related to the generation of aura before migraine attack; (3) activating the trigeminal vascular system; and (4) promoting the release of CGRP, Substance P (SP), and other neurotransmitters. In other words, Glu plays an important role in migraine with a great potential for drug discovery [22].

Multiple physiological activities in multiple brain regions participate in the onset of migraine, the necessary part being the activation of the trigeminal system. As a central projection termination zone for trigeminal nerves in the cervical nerve, trigeminocervical complex (TCC) can transmit stimulation to trigeminoaminoparasympathetic nerves upwards and transmit nociceptive information from the head and viscera to hypothalamus, thalamus downwards. In addition, the direct connection of TCC to the brainstem region (including midbrain periaqueductal gray, nucleus raphe nucleus, and blue patch) makes it as a hub for regulating the transmission of nociceptive stimuli [23, 24].

Here, we studied the effect of DCXF in the NTG-induced migraine rat, explored potential amino acid biomarkers by metabolomics using GC-TOF-MS, and fouced on the glutame-glutamine cycle, aiming to provide references for the understanding of the mechanism of DCXF.

## 2. Methodology

### 2.1. Chemicals and Reagents

The active components extracted from Gastrodia (contained 5.46% of gastrodin) and Chuanxiong (contained 3.74% of ferulic acid) were obtained as the previous reported [25, 26] (for more details in [Supplementary-material supplementary-material-1]). Nitroglycerin (NTG) and flunarizine hydrochloride capsules (FHC) were purchased from Xian Janssen Pharmaceutical Ltd. (Xian, China) and Beijing Yimin Pharmaceutical Co., Ltd. (Peking, China), respectively. Pyridine, anhydrous ethanol, and chloroform were of analytical grade from China National Pharmaceutical Group Corporation (Shanghai, China). The ultrapure water was made by the Milli-Q system (Millipore, USA). Methoxyamine, L-2-chlorophenylalanine (an internal quality standard), and N, O-bis (trimethylsilyl)trifluoroacetamide (BSTFA), containing 1% trimethylchlorosilane (TMCS), were purchased from Sigma-Aldrich (St. Louis, MO, USA). Trizol reagent was obtained from Life Technology (CA, USA). PrimeScript® RT Master Mix and SYBR® Premix Ex Taq™ were purchased from Applied Biosystems (Foster City, CA). The primer sequences were designed by Shanghai Generay Biotech Co., Ltd. (Shanghai, China). Glyceraldehyde-3-phosphate dehydrogenase (GAPDH) antibody was purchased from Servicebio (Wuhan, China). GS antibody was obtained from Invitrogen (CA, USA). Secondary antibodies, BCA kit, and RIPA lysis buffer were from Beyotime (Jiangsu, China).

### 2.2. Preparation of Drugs

The DCXF, composed by 7.60 g extraction of Gastrodia and 5.24 g extraction of chuanxiong, was dissolved in physiological saline and diluted to form the solution including 8.30 mg/mL of gastrodinand 3.92 mg/mL of ferulic acid (details was provided in [Supplementary-material supplementary-material-1]). The FHC (each capsule contains 5 mg flunarizine hydrochloride) prepared in physiological saline and diluted to 0.125 mg/mL FHC solution was used as the positive drug.

All drug solutions were stored at 4°C and shook well before use.

### 2.3. Animal

Sixty male Sprague Dawley rats (170–210 g) were supplied by the Lab Animal Center of Shanghai University of Traditional Chinese Medicine (SCXK(Hu)2013-0016). They were maintained in an environmentally controlled breeding room (22 ± 0.8°C, 50∼60% relative humidity, and 12 h/12 h light/dark cycle) with food and water freely available for 1 week before starting the experiments. The animal facilities and protocols were approved by the Institutional Animal Care and Use Committee, Shanghai University of Traditional Chinese Medicineon4^th^ Sep, 2016 (no. SZY201609010). All procedures were conducted in accordance with the Guide for the Care and Use of Laboratory Animal (the National Academies Press, revised edition 2010).

### 2.4. Drug Administration and Sampling

All the rats were randomly divided into 4 groups, named the normal group (*n* = 6), model group (*n* = 18), DCXF group (*n* = 18), and FHC group (*n* = 18). Except the normal group, other groups of rats were subcutaneously injected with 10 mg/kg of NTG to establish an experimental rat migraine model as reported by Tassorelli et al. [27]. The model group was sorted into three subgroups (each subgroup contained 6 rats, named the M30 group, M60 group, and M90 group) by the sampling time for collecting blood after final operation for 30 min, 60 min, and 90 min. As for the DCXF group and FHC group, the corresponding drug solutions were orally administered to rats at a dosage of 10 mL/kg after 30 minutes, respectively. Then, the DCXF group and FHC group were as same as the model group to the classification of subgroups. The TCC part was rapidly removed after collecting blood. All the samples were stored at −80°C.

### 2.5. Analytical Methods

#### 2.5.1. GC-TOF-MS

For GC-TOF-MS analysis, the procedure was performed as reported by Chen-Tian Shen with minor adjustment [28]. The sample of the TCC issue was homogenized in ice cold water (2.5 mL/g). 250 *μ*L chloroform-methanol (1 : 3, v/v) as well as 10 *μ*L L-2-chlorphenylalanine aqueous solution was added to the 500 *μ*L homogenate and vortexed for 1 min before keeping at −20°C for 20 min. As for the serum samples, 250 *μ*L chloroform-methanol solvent was mixed with 100 *μ*L serum. After centrifugation at 12000r min^−1^ for 10 min at 4°C, 300 *μ*L of supernatant was obtained and concentrated in a vacuum (about 2 h to 2.5 h) and then again nitrogen-dried. 80 *μ*L of methoxyamine (15 mg/mL in pyridine) was quickly added and sealed to performmethoxymation at 37°C for 90 min. Then, 80 *μ*L of BSTFA was added quickly and sealed to react at 70°C for 60 min for trimethysilylation.

The sample injection volume was 1.0 *μ*L, and the injection temperature was 280°C. A DB-5MS column (5% phenyl-95% dimethylpolysiloxane) was used to separate the sample. The column temperature was set at 90°C for 0.2 minutes, raised to 180°C °C at 10°C/min, raised to 240°C at 5°C/min, raised to 300°C at 25°C/min, and finally held at 300°C for 10.0 min. The superpure helium (≥99.9999%)was used as carrier gas at a constant flow rate of 1.0 mL/min. The temperature of the transfer line and ion source was set at 270 and 220°C, respectively. The full scan mode (*m*/*z* 30–600) was used to acquire the data. The serum sample solvent time delay was 7.5 min; the brain tissue sample solvent time delay was 7.6 min.

#### 2.5.2. RT-PCR

The total mRNA was isolated by the Trizol method. Then, they were dissolved in sterile distilled water and quantified by optical density at 260 nm and 280 nm. cDNA was synthesized by reverse transcription according to the Kit instructions in PrimeScript® RT Master Mix. Real-time PCR was performed by using a SYBR green premix according to the kit method. GAPDH was used as an internal control. Data were presented according to the 2^−△△Ct^ method. The primer sequences used in this section were shown in [Supplementary-material supplementary-material-1].

#### 2.5.3. Western Blotting

The total proteins from TCC tissues were extracted by sonication in RIPA lysis buffer with phenylmethanesulfonyl fluoride (PMSF). Following centrifugation at 12,000 rpm for 10 min at 4°C, the supernatant of the lysate was collected and analyzed for protein content by using a BCA protein assay kit. The amounts of proteins used for western blot analysis were 40 *μ*g to detect GAPDH and GS. The proteins were loaded onto 4–12% SDS-PAGE gels and transferred onto NC membranes. Then, the NC membranes were placed in 0.1% formaldehyde solution for 30 min in order to fix protein. The membranes were saturated in tris-buffered saline with Tween-20 (TBST) with 5% nonfat-milk for 1 h at room temperature and then incubated with anti-GAPDH and anti-GS overnight at 4°C. Washed by TBST, the blots were incubated with secondary antibodies for 40 min. Finally, the bands were visualized with the ECL system. The GS contents were normalized using GAPDH.

### 2.6. Statistical Analysis

For GC-TOF-MS analysis, raw data were exported into the CDF format by Chroma TOF software (v 3.30; Leco Co.). After preprocessing, data that contained sample information, peak retention, and peak area were obtained. Statistical analysis was performed by Simca-P 11 software.

The variable importance in the projection (VIP) values of all data from the OPLS model and the significance of each metabolite variance measured by the Student's test were considered as a coefficient for differentiating variables. A list of metabolites that had influence on the variance (VIP > 1 and *P* < 0.05) were chosen for identification and further analysis.

For the Real-time PCR, all data were expressed as mean ± SD. If data were normally distributed and samples were independent, then *T*-tests were used for two-group comparisons while one-way analysis of variance was used to compare multiple groups; if not, nonparametric tests were used, including the Wilcoxon rank sum test and the Kruskal–Wallis test.

Statistical significance was accepted at *P* < 0.05.

## 3. Results

### 3.1. Metabolomics Study

#### 3.1.1. Overall Metabolic Differences by GC-TOF-MS

**Figure 1:**
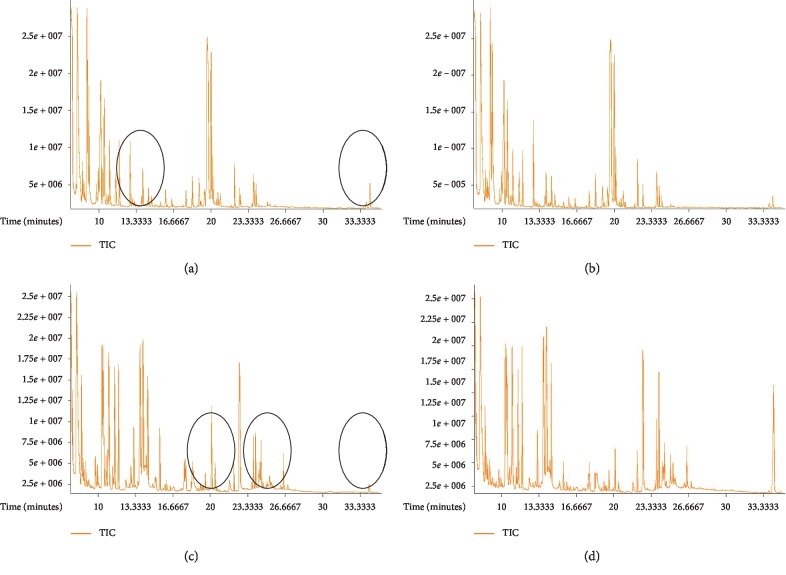
The GC-TOF-MS fingerprinting of (a) the control group from serum; (b) the M90 group from serum; (c) the control group from the TCC sample; and (d) the M90 group from the TCC sample.

Typical total ion chromatograms (TIC) of serum and brain tissue samples from the control group and M90 group are illustrated in Figure 1: serum (a) for the control group and (b) for the M90 group and the TCC sample (c) for the control group and (d) for the M90 group. Obvious difference among the four groups could be visually inspected, which suggested that modelling and administration could change the metabolic status (The area is marked by the black circle for easily comprehension).

#### 3.1.2. Different Metabolism in the Control Group, M90 Group, D90 Group, and F90 Group

**Figure 2:**
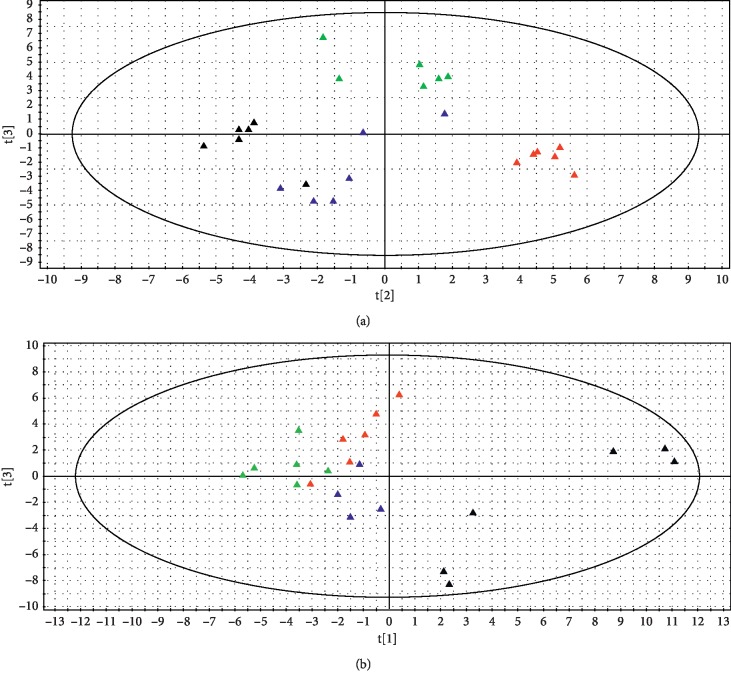
Score plot of the control group (black triangle), M90 group (red triangle), D90 group (deep blue triangle), and F90 group (green triangle) from a PLS-DA model of serum (a) and brain tissue (b).

With the aim to more clearly distinguish for the overall metabolic differences among the groups, PLS-DA was carried out, resulting in Figure 2: serum (a) and TCC sample (b).

As observed from Figure 2, the distinct clustering and clear separation of the four groups were obtained without overlap in serum. The separation between the control group and model group suggested that biochemical perturbation occurred after modelling by NTG. Compared with the model group, the DCXF group was much closer to the control group, suggesting that DCXF might help to relieve migraine by improving the metabolic disorders. At the same time, the FHC group was also close to the control group but did not coincide with the DCXF group, which could be indicative of a different mechanism for the two drugs.

In the brain tissue sample, there was little overlap with the M90 group and F90 group. Compared with the control group, the D90 group was closer to the M90 group. Despite of it, the D90 group still showed the tendency to the the control group.

#### 3.1.3. Time-Dependent Metabolic Trajectory in the Model Group, DCXF Group, and FHC Group

**Figure 3:**
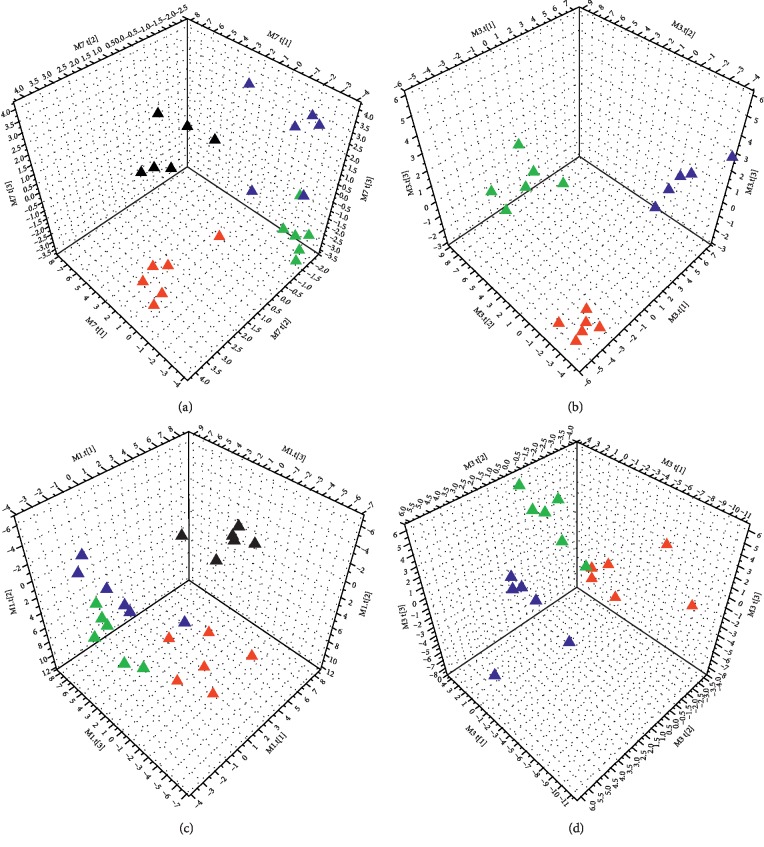
Score plot from a PLS-DA model of (a) the model group from serum; (b) the DCXF group from serum sample; (c) the model group from the TCC sample; and (d) the DCXF group from the TCC sample. The black triangle in a and b was the control group. red triangle for the 30 min subgroup; deep blue triangle for the 60 min subgroup; green triangle for the 90 min subgroup; and brown triangle for the 120 min subgroup.

In the interest of observing the time-dependent metabolic trajectory, three subgroups that are divided by different sampling time of the model group and DCXF group were analyzed by PLS-DA. Results are showed in Figure 3: serum (a) for the model group and (b) for the DCXF group and TCC sample (c) for the model group and (d) for the DCXF group.

As we could see from Figures 3(a) and 3(c), the spots of the model group were clearly separated from the control group from 30 min to 90 min, suggesting that the major changes happened in the metabolic network from the control group, both in serum and brain tissue.

The serum sample that are collected at different time periods from the DCXF group (Figure 3 b) showed obvious separation and time tendency, indicating that the DCXF could change the metabolic status in serum of migraine rats induced by NTG with the increasing treatment time. Although the D60 group in the brain tissue did not cluster well, it still could be differentiated with the D90 group and D120 group.

To sum up, the different groups at different time were generally distinguished by the PLS-DA and showed time tendency to varying degree.

#### 3.1.4. Analysis and Identification of Potential Biomarkers

*(1) Potential Biomarkers Responsible for Migraine Induced by NTG*. The matabolites with VIP > 1 and *P* < 0.05 (by *T*-test) were chosen as potential biomarkers. Then, NIST and the database established by Analysis and Testing Center of Shanghai Jiao Tong University were used for identification. Twenty-one metabolites in the serum and 18 metabolites in the brain tissue were considered as potential biomarkers related to tell the difference between the control group and the M90 group (shown in Table 1).

*(2) Potential Biomarkers Responsible for the Antimigraine Effect of DCXF and FHC*. Similar processes were carried out to get the potential biomarkers for the antimigraine effect of DCXF and FHC. In the end, 9 common potential biomarkers in the serum and 6 common potential biomarkers in the brain for DCXF and FHC were obtained, respectively. Besides the common potential biomarkers, it might help to understand the different mechanisms for the two drugs by characteristic potential biomarkers. The 4 characteristic metabolites of DCXF in the serum included carnitine, capric acid, 3-hydroxyphenylacetic acid, and linoleic acid, while the 8 characteristic metabolites of FHC consisted of ribitol, 2(alpha-D-mannosyl)-D-glycerate, arachidonic acid, and so on. In the brain, there were 5 characteristic potential biomarkers of DCXF and 6 of FHC, no longer repeating. The results are summarized in Table 1.

In addition, it was not difficult to find out the DCXF could reverse the levels of the potential biomarkers that are responsible for its antimigraine effect. From Table 1, there were 21 metabolites changed in the serum when the M90 group was compared with the control group, but DCXF could reserve 8 metabolites to their levels in the control group (*P* > 0.05). Similar tendency was shown in the brain tissue. In other words, DCXF could change the concentrations of the potential biomarkers to have the tendency to the control group to a certain degree.

#### 3.1.5. Possible Pathways

With the purpose to explore the possible metabolic pathways involved in the therapeutic mechanism, the metabolites that contributed to the change of the metabolic state resulting from DCXF were imported to the MetaboAnalyst 3.5 (Shown in Table 2). Alanine, aspartate and glutamate metabolism, galactose metabolism, and lysine degradation were common pathways for DCXF in the serum and TCC sample. Meanwhile, fatty acid metabolism was also involved in metabolic state changes, such as the biosynthesis of unsaturated fatty acids in serum and the metabolism of propionate in the TCC sample. Differences in potential pathways obtained from the serum and TCC sample might lead to different mechanisms, which could provide crucial information for understanding the mechanisms associated with DCXF treatment.

### 3.2. Changes in Expression of Genes Involved in the Glutamate Metabolism by DCXF Treatment

**Figure 4:**
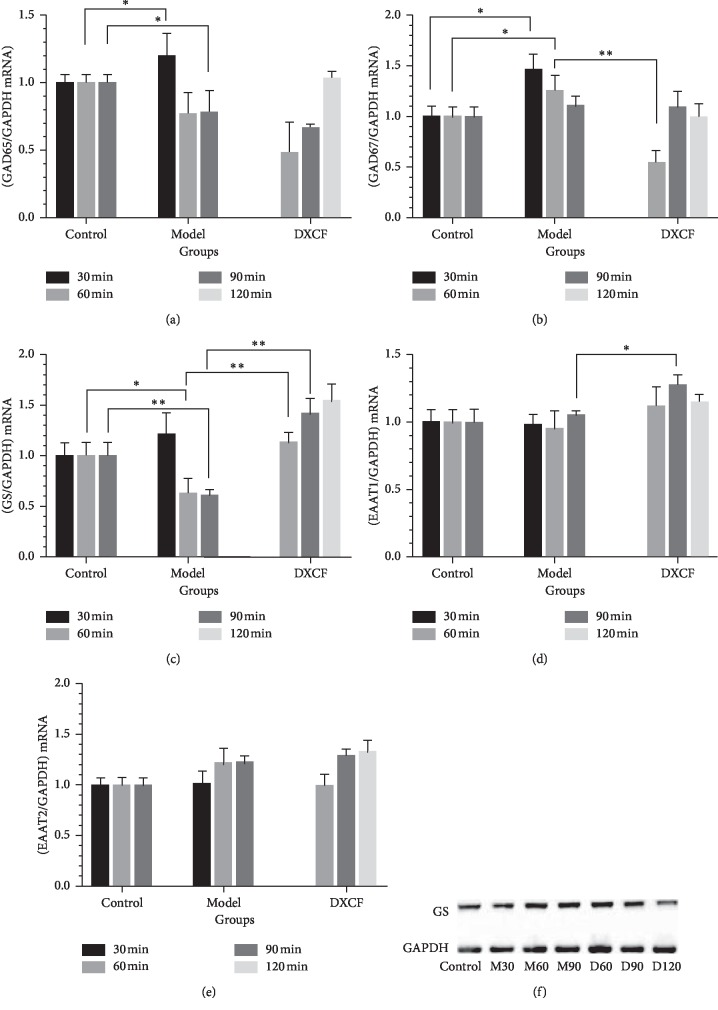
The mRNA expressions of GAD65, GAD67, GS, EAAT1, and EAAT2(A-E). Data were expressed as mean ± SD (*n* = 6). ^*∗*^*P* < 0.05, ^*∗∗*^*P* < 0.01; the expression of GS protein (F).

Based on the results of the possible pathways and the related articles [22], the glutamate metabolism was paid close attention. The mRNA expressions of GAD65, GAD67, GS, EAAT1, and EAAT2 were observed, all of which were involved in the transport of glutamate cycles (shown in Figures 4(a)–4(e)).

GAD is an important rate-limiting enzyme for *γ-*aminobutyric acid (GABA)synthesis, and it has two subtypes sorted by different molecular weights, namely GAD65 and GAD67. The results showed that the expressions of GAD65 in the M60 group and M90 group were lower than those of the control group (both *P* < 0.05). However, there was no difference by comparing the DCXF group. As for the expression of GAD67, the increasing tendencies could be detected in the M30 group and M60 group compared with the control group, and the D60 group had significant difference with the model group.

GS plays an important role in the glutamate-glutamine cycle by transforming glutamate into glutamine. Compared with the control group, the expressions of GS in the M30 group and M60 group were downregulated (*P* < 0.05, *P* < 0.01). After given DCXF, GS in the D60 group was lower than that in the M60 group (*P* < 0.01).

However, the changes of EAAT1 and EAAT2 that took part in the transport of glutamate were not significant. There was no difference between the model group and the control group. We could only see that the expression of EAAT1 of the D90 group was higher than that of the M90 group (both *P* < 0.05).

### 3.3. The Decreased Tendency of the GS Protein Expression by DCXF Treatment

In an attempt to identify the change of the GS, western blot was used to measure the protein levels of GS to observe the effect of DCXF on migraine rats (Figure 4(f)). However, the expression of GS was unchanged with the drug treatment, compared with the model group. Perhaps the change of protein level needs more time than that of mRNA.

## 4. Discussion

We confirmed that DCXF could improve the abnormal metabolic state in the serum and TCC sample of migraine rats caused by the NTG and have shown the time-dependent tendency of the efficacy of DCXF for the first time. Additionally, it seemed that the glutamine metabolism could account for antimigraine effect of DCXF (we also provided the results about the antimigraine effect of DCXF by animal behavior and immunohistochemical section in [Supplementary-material supplementary-material-1]).

Firstly, we explored the metabolic pathways of the model group and the DCXF group at different time periods to determine if the changes in the metabolism profile were time dependent. From Figures 3(a) and 3(b), major changes happened in the metabolic network after giving NTG over time. The different subgroups of the DCXF group were well differentiated and showed time-dependent trends. The samples of the D120 group and D60 group were not coincident, indicating that the metabolic state of the serum and TCC sample in rats changed continuously after DCXF administration.

Secondly, results from Figure 2 showed that DCXF changed the metabolic states in the TCC sample and serum of the migraine rats induced by NTG after modeling 90 min to make it close to the control group. Taking into account that migraine was a neurological disease and amino acids played an important role in migraine, the metabolic changes associated with amino acids in the TCC sample were analyzed particularly in this paper.

The level of succinic acid in the M90 group at the TCC sample increased, probably caused by the conversion of glutamate to *α*-ketoglutarate, which then entered the tricarboxylic acid cycle to synthesize succinic acid. DCXF reduced the concentration of succinic acid by converting glutamic acid to glutamine under the catalytic action of GS probably. Alanine, aspartate metabolism, and lysine degradation were also involved in the regulation of migraine by DCXF. Aspartic acid can be introduced into the tricarboxylic acid cycle by the formation of fumarate or oxaloacetate, and the next product of oxaloacetate is citric acid. The citric acid content in the M90 group was increased, possibly due to the increased metabolism of aspartic acid. Aspartic acid is an excitatory neurotransmitter. Studies have shown that patients with chronic migraine had altered interregional N-acetyl-aspartate correlations, compared with the healthy controls [29]. We speculated that the metabolic abnormality of aspartic acid might contribute to the onset of migraine attacks. DCXF could downregulate the level of citric acid compared with the control group without being statistically different. Also, citric acid was a characteristic potential metabolite of the DCXF group, which differentiates the effect of DCXF and FHC on aspartic acid to a certain degree.

Thirdly, we could get more support to the glutamate metabolism that played an important role in migraine induced by NTG from the results of mRNA and protein expressions.

In the glutamate cycle, GS can catalyze glutamate to produce glutamine. The RNA expression of GS in the model group was lower than that in the control group, especially the M90 group and M60 group, indicating that the hinderance that is caused by the NTG might exist in the transformation of glutamate to glutamine. After given DCXF, the increased RNA expression of GS and subsequent metabolism might result in less glutamate and more glutamine. Then, the toxicity of glutamate for neurons could be reduced to improve migraine attack. Also, Bathel et al. have found the increased thalamic glutamate/glutamine levels in migraineurs [30]. However, taking into account that the level of GS protein was unchanged, further experiment should be processed.

**Figure 5:**
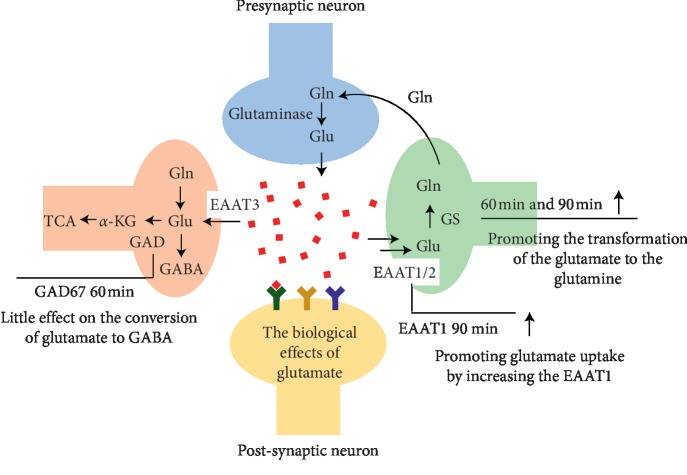
The results of the glutamate cycle by DCXF.

EAAT1 and EAAT2 are essential in preventing excitotoxicity, which can be modulated by drugs at multiple levels of gene expression [31], and functional upregulation of glutamate transporters is turning into a prospective pharmacotherapeutic approach for the management of chronic pain [32]. As for DCXF, the results showed that there is no significance in the expressions of EAA1 and EAAT2, indicating the DCXF may have little influence on this in the TCC. Considering that EAAT4 was only detected in the dendrites of cerebellar Purkinje neurons and EAAT5 was in the retinal may be the expression of EAAT3 needed to be supplemented in the following study. In addition, the expression of GAD65 in the model group decreased at 60 min and 90 min after modelling, and GAD67 expression decreased at 30 min and 60 min after modelling. Glu can be transformed into GABA by GAD65/GAD67, and GABA was considered as a diagnostic marker potentially in migraine pathophysiology [33]. Given this, we hypothesize that the conversion of glutamic acid to GABA may be hindered and the imbalance between the excitatory amino acids and inhibitory amino acids was exacerbated by NTG. But, the expression of GAD67 in the DCXF group decreased at 60 min. It might be that DCXF had little effect on the conversion of glutamate to GABA. Similarly, DCXF had no effect on GAD65 (shown in Figure 5). There remains much doubt about the link between the DCXF and GAD65/GAD7, and more data are needed.

## 5. Conclusion

DCXF could change the metabolic state of migraine rats induced by the NTG in serum and TCC sample so as to improve the migraine, showing the time-dependent tendency. The amino acid metabolism (glutamate, aspartate and alanine metabolism, and lysine degradation) and galactose metabolism, linoleic acid metabolism, and pentose phosphate pathway were possibly involved in the mechanism of DCXF treatment. The study about the glutamate cycle indicated that DCXF could improve the glutamate metabolism by increasing the GS and EAAT1 to promote the conversion of glutamate to glutamine as well as the glutamate uptake.

## Figures and Tables

**Table 1:** Summary of potential biomarkers.

Sample	Sort	Metabolites	Fold change M90/C	Fold change D90/C	Fold change F90/C
Serum	Common potential biomarkers	Isopentenyl pyrophosphate	2.3^*∗∗*^	1.05^##^	1.34^##^
		L-Asparagine	0.65^*∗*^	1.42^##^	1.3^##^
		Aminomalonic acid	0.59^*∗∗*^	1.1^##^	0.98^##^
		21-Hydroxypregnenolone	37.92^*∗∗*^	288.2^*##∗∗*^	152.31^*##∗∗*^
		Fluorene	0.51^*∗∗*^	1.06^##^	0.96^##^
		5-Methoxytryptamine	0.69^*∗*^	2.41^*##∗∗*^	1.72^*##∗∗*^
		Beta-glycerophosphoric acid	0.59^*∗*^	1.2^#^	1.47^##^
		Farnesal	0.49^*∗∗*^	1.07^#^	0.78^##^
		Alpha-lactose	0.23^*∗∗*^	0.45^*#∗*^	0.55^##^
	Characteristic potential biomarkers for DCXF	Carnitine	1.69^*∗*^	3.05^*#∗∗*^	1.66
		Capric acid	1.81^*∗*^	0.54^#^	1.1
		3-Hydroxyphenylacetic acid	1.6^*∗*^	5.856^*#∗∗*^	3.78
		Linoleic acid	0.48^*∗*^	1.04^#^	0.55
	Characteristic potential biomarkers for FHC	Ribitol	1.46^*∗*^	2.45	2.3^*##∗∗*^
		2(alpha-D-Mannosyl)-D-glycerate	4.44^*∗∗*^	157.23	8.11^*#∗∗*^
		Arachidonic acid	0.6^*∗∗*^	0.67	0.94^##^
		Palmitic acid	0.44^*∗∗*^	0.56	0.68^*#∗*^
		Allose	0.59^*∗∗*^	0.95	1.08^##^
		Beta-glycerophosphoric acid	0.57^*∗*^	0.77	0.96^#^
		Alpha-tocopherol	0.35^*∗*^	0.46	0.88^##^
		Glycerol	0.5^*∗∗*^	0.61	1.06^##^

Brain tissue	Common potential biomarkers	Phosphoglycolic acid	1.62^*∗∗*^	1.21^#^	1.04^#^
		D-erythrose 4-phosphate	1.56^*∗∗*^	1.17^#^	0.99^##^
		Erythrose	1.40^*∗∗*^	0.97^#^	1.99^*##∗∗*^
		Inosine	1.64^*∗*^	1.10^##^	0.95^##^
		3,3′,4′5-Tetrahydroxystilbene	1.52^*∗∗*^	0.68^#^	2.18^*#∗∗*^
		Hydroxyatrazine	1.65^*∗∗*^	1.16^##^	0.53^##^
	Characteristic potential biomarkers for DCXF	Carnitine	1.89^*∗∗*^	1.34^##^	2.56
		Fluorene	1.80^*∗*^	1.27^##^	2.24
		Citric acid	1.61^*∗∗*^	1.05^##^	1.23
		Succinic acid	1.38^*∗∗*^	1.11^##^	1.61
		Adenine	1.43^*∗∗*^	1.18^#^	1.46
	Characteristic potential biomarkers for FHC	Glyceric acid	1.76^*∗∗*^	1.58	0.46^*##∗∗*^
		D-glucose	0.71^*∗*^	0.7	1.04^#^
		N-formyl-L-methionine	1.60^*∗∗*^	1.29	1.17^##^
		Alpha-linolenic acid	2.11^*∗*^	1.97	1.21^#^
		Trehalose 6-phosphate	1.83^*∗∗*^	1.58	1.22^#^
		Prostaglandin A2	6.74^*∗*^	6.99	1.14^#^

Compared with the control group, ^*∗∗*^*P* < 0.01, ^*∗*^*P* < 0.05; compared with the M90 group, ^##^*P* < 0.01, ^#^*P* < 0.05.

**Table 2:** Possible pathways for DCXF.

Serum	Brain
Terpenoid backbone biosynthesis	Galactose metabolism
Alanine, aspartate, and glutamate metabolism	Glyoxylate and dicarboxylate metabolism
Aminoacyl-tRNA biosynthesis	Pentose phosphate pathway
Steroid hormone biosynthesis	Purine metabolism
Galactose metabolism	Alanine, aspartate, and glutamate metabolism
Lysine degradation	Lysine degradation
Biosynthesis of unsaturated fatty acids	Propanoate metabolism
Linoleic acid metabolism	Citrate cycle (TCA cycle)

## Data Availability

The data used to support the findings of this study are available from the corresponding author upon request.

## Abbreviation

5-HT: 5-Hydroxytryptamine
BSTFA: Bis(trimethylsilyl)trifluoroacetamide
CGRP: Calcitonin gene-related peptide
CSD: Cortical spreading depression
DCXF: Active components extracted from dachuanxiong prescription
EAAT-1: Excitatory amino acid transporter-1
EAAT-2: Excitatory amino acid transporter-2
FHC: Flunarizine hydrochloride capsules
GABA: *γ*-aminobutyric acid
GAD: Glutamate decarboxylase
GAPDH: Glyceraldehyde-3-phosphate dehydrogenase
GS: Glutamine synthetase
NTG: Nitroglycerin
OPLS-DA: Orthogonal partial least squares discriminant analysis
PCA: Principal component analysis
PLS-DA: Partial least squares-discriminate analysis
PMSF: Phenylmethylsulfonyl fluoride
TBST: Tris buffered saline with Tween-20
TCC: Trigeminocervical complex.

## Supplementary Materials

Supplementary Materials Supplementary Table ST1: the determination of the ferulic acid in the active components extracted from chuanxiong and the gastrodin extracted from gastrodia, providing more details about the drugs used in this research paper. Supplementary Table ST2: sequences of primers used for real-time PCR. Supplementary Figure S1: animal behaviors after giving DCXF on the migraine rats. Supplementary Figure S2: the expression of CGRP in the brain tissue of DCXF by immunohistochemical section.
