# Supplementary material for: Studies on the Mechanism of Glutamate Metabolism in NTG-Induced Migraine Rats Treated with DCXF
Source: Evid Based Complement Alternat Med. 2019 Dec 31;2019:1324797. doi: 10.1155/2019/1324797 (PMC7011483; doi:10.1155/2019/1324797)
Supplement: Supplementary Materials — Supplementary Table ST1: the determination of the ferulic acid in the active components extracted from chuanxiong and the gastrodin extracted from gastrodia, providing more details about the drugs used in this research paper. Supplementary Table ST2: sequences of primers used for real-time PCR. Supplementary Figure S1: animal behaviors after giving DCXF on the migraine rats. Supplementary Figure S2: the expression of CGRP in the brain tissue of DCXF by immunohistochemical section. [file 1324797.f1.docx]

**Supplement 1**

Active components extracted from Chuanxiong:

The roots of *Ligusticumchuanxiong Hort.* were purchased from Shanghai Kangqiao Chinese Medicine Tablet Co., LTD. and was previously identified by Prof. Zhili Zhao at Shanghai University of Traditional Chinese Medicine. The ferulic acid (110773-201313) was got from China Institute of food and drug control. Other reagents were purchased from Sinopharm Group Chemical Reagent Co., Ltd.

The power of Chuanxiong were soaked with 70% ethanol (12 times volume).After reflux extraction for 3 times, all the filtrate was collected and mixed to recycle ethanol to a concentration of 0.5 g·mL^-1^ crude drug. The concentrate was centrifuged at 5000 r·min^-1^ for 15 min. HPD-100 macroporous resin was used to separate the supernatant by 1 BV·h^-1^ of the water. Then 50% ethanol would replace the water till the eluent was near colorless and the ethanol eluate was collected. Finally, active components extracted from Chuanxiong were got after recover the ethanol and vacuum drying. In order to make sure of the content of the ferulic acid, HPLC was applied and results were shown in Table 1. The ferulic acid mass fraction is 3.74%.

Active components extracted from Gastrodia:

The roots of*Gsatrodiaelata BL.* were purchased from Shanghai Kangqiao Chinese Medicine Tablet Co., LTD. and was previously identified by Prof. Zhili Zhao at Shanghai University of Traditional Chinese Medicine. The Gastrodin (110807-201507) was got from China Institute of food and drug control. Other reagents were purchased from Sinopharm Group Chemical Reagent Co., Ltd.

The power of Gastrodia were soaked with 70% ethanol (10 times volume). After reflux extraction for 3 times, all the filtrate was collected and mixed to recycle ethanol to a concentration of 0.5 g·mL^-1^ crude drug. The concentrate was centrifuged at 5000 r·min^-1^ for 15 min. Then the supernatant was collected and concentrated to a concentration of 4 g·mL^-1^ crude drug. Ethanol was added to the concentrate till the content of ethanol was 70%. After placing for 24 h, the extract was filtered and NaOH was added to adjust the filter to PH 8.0. Go on placing for 24 h, active components extracted from Gastrodia were got after recover the ethanol and vacuum drying. The content of gastrodin was also tested by HPLC (Shown in ST2). The gastrodin mass fraction is 5.30%.

ST1. Determination of ferulic acid and gastrodin of active compoments

| Standard | 20160723  （mg·g^-1^） | 20161014  （mg·g^-1^） | 20170411  （mg·g^-1^） | 20170628  （mg·g^-1^） | Average  （mg·g^-1^） | RSD（%） |
| --- | --- | --- | --- | --- | --- | --- |
| Ferulic acid | 36.56 | 39.04 | 35.31 | 38.86 | 37.44 | 1.82 |
| Gastrodin | 52.59 | 55.92 | 53.72 | 56.11 | 54.58 | 1.72 |

For more details, please reference the following paper.

Yanlong Hong, Desheng Xu, Yi Feng, et al., 2008. Study on extraction and purification of Gastrodia extract. Chinese Tradit. Pat. Med. 36, 204–207. https://doi.org/10.1093/nar/gkn542

Yanlong Hong, Desheng Xu, Yi Feng, et al., 2007. Study on extraction and purification of active parts of ferulic acid from Ligusticum chuanxiong. China J. Chinese Mater. Medica 1740–1743.

Yanlong Hong, Yi Feng, Desheng Xu, et al., 2007. Study on extraction and purification of active parts from Da Chuan Xiong Fang for treatment of migraine. J. Chinese Med. Mater. 30, 721–723.

Qingqing Wang, Lan Shen, Jun Zhang, et al., 2018. Effects of the active components extracted from Da Chuanxiong Fang on the expression of CGRP and its receptors in rats with migraine. J. Chinses Traditional Patent Medicine. 40, 14-20.

**Supplement 2**

ST2 Sequences of primers used for real-time PCR

| Primers | Sequence-Forward | Sequence-Reverse |
| --- | --- | --- |
| GS | AGGAGAATGGTCTGAGGTG | AGTCGTTGATGTTGGAGGT |
| GAD65 | TCTTTTCTCCTGGTGGTGCC | CCCCAAGCAGCATCCACAT |
| GAD67 | CTGCTCCAGTGTTCTGCCATCCT | CCCTCGGAGGCTTTGTGGTATG |
| EAAT-1 | ATACAACCAAGGCAGTCATC | CGAAAGCAATAAAGAATCCA |
| EAAT-2 | ATACAACCAAGGCAGTCATC | CGAAAGCAATAAAGAATCCA |
| GAPDH | TGATTCTACCCACGGCAAGTT | TGATGGGTTTCCCATTGATGA |

**Supplement 3**

Results about the anti-migraine effect of DCXF

Animal behavior

After subcutaneous injection of nitroglycerin, the rats in the model group showed behaviors such as scratching, climbing, and tail biting. Given FHC or DCXF, the activity of the rats decreased, showing a quiet statue (Shown in Figure S1).


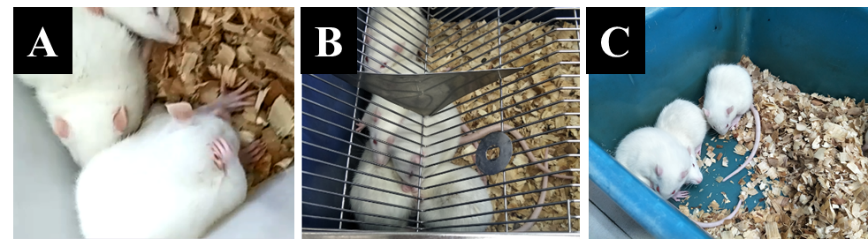


Figure S1. Behavior after modeling by nitroglycerin; (A) M90 group;(B) F60 group;(C) D60 group

Immunohistochemical section

The expression of CGRP in the brain tissue of each group was observed under a microscope of 400 times. The results were shown in Figure S2. CGRP-positive cells appeared brownish yellow, mostly near-circular. Compared with the normal group, the expression of CGRP in the subgroup of the model group increased significantly, suggesting that an acute migraine model induced by nitroglycerin has been established. After administration of FHC or DCXF, the expression of CGRP was decreased, indicating that the drug has the anti-migraine effect by reducing the expression of CGRP.


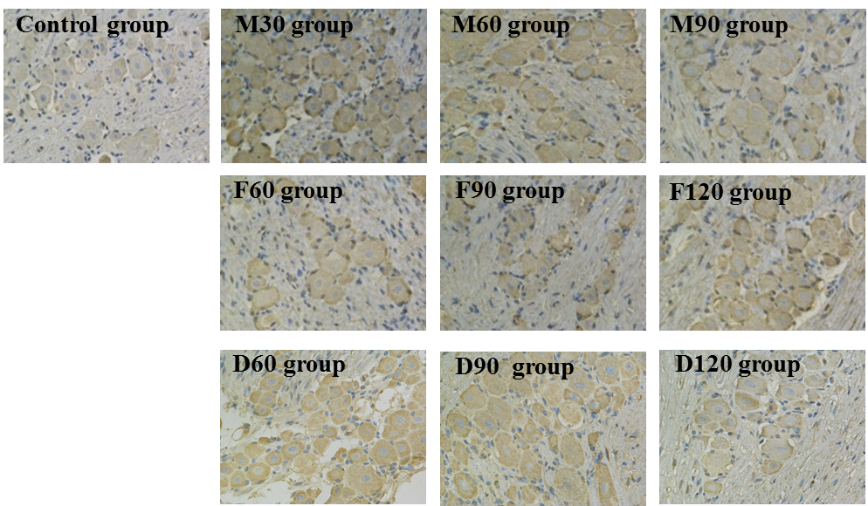


Figure S2. Expression of CGRP in TCC by immunohistochemical staining(×400)
